# Interplay between ultrastructural findings and atherothrombotic complications in type 2 diabetes mellitus

**DOI:** 10.1186/s12933-015-0261-9

**Published:** 2015-07-31

**Authors:** Prashilla Soma, Etheresia Pretorius

**Affiliations:** Department of Physiology, Faculty of Health Sciences, University of Pretoria, Pretoria, South Africa

**Keywords:** Diabetes, Morphology, Platelets, Fibrin, Erythrocytes, Atherosclerosis

## Abstract

Accelerated atherosclerosis is the main underlying factor contributing to the high risk of atherothrombotic events in patients with diabetes mellitus and atherothrombotic complications are the main cause of mortality. Like with many bodily systems, pathology is observed when the normal processes are exaggerated or uncontrolled. This applies to the processes of coagulation and thrombosis as well. In diabetes, in fact, the balance between prothrombotic and fibrinolytic factors is impaired and thus the scale is tipped towards a prothrombotic and hypofibrinolytic milieu, which in association with the vascular changes accompanying plaque formation and ruptures, increases the prevalence of ischaemic events such as angina and myocardial infarction. Apart from traditional, modifiable risk factors for cardiovascular disease like hypertension, smoking, elevated cholesterol; rheological properties, endogenous fibrinolysis and impaired platelet activity are rapidly gaining significance in the pathogenesis of atherosclerosis especially in diabetic subjects. Blood clot formation represents the last step in the athero-thrombotic process, and the structure of the fibrin network has a role in determining predisposition to cardiovascular disease. It is no surprise that just like platelets and fibrin networks, erythrocytes have been shown to play a role in coagulation as well. This is in striking contrast to their traditional physiological role of oxygen transport. In fact, emerging evidence suggests that erythrocytes enhance functional coagulation properties and platelet aggregation. Among the spectrum of haematological abnormalities in diabetes, erythrocyte aggregation and decreased deformability of erythrocytes predominate. More importantly, they are implicated in the pathogenesis of microvascular complications of diabetes. The morphology of platelets, fibrin networks and erythrocytes are thus essential role players in unravelling the pathogenesis of cardiovascular complications in diabetic subjects.

## Background

The trend regarding the incidence of type 2 diabetes mellitus is that it is increasing in the general population because of increasing obesity and is likely to subsequently increase the incidence of coronary artery disease. It is also known that risk factors such as obesity, hypertension and hypercholesterolemia are crucial to the development of atherosclerosis which results in inflammation [1]. Fundamental to contributory factors of morbidity and mortality in diabetes is atherothrombotic complications [2]. Despite advances in antiplatelet therapies and control of modifiable risk factors; like hypertension, obesity, smoking and dyslipidaemia, the risk of ischaemic events remains high in patients with type 2 diabetes. There is thus a pressing need to understand the complexity of mechanisms contributing to atherothrombotic complications so that more effective therapies can be developed. Evidence shows that diabetes has been considered to be a prothrombotic status. Characteristic findings in type 2 diabetes includes: increased coagulation, impaired fibrinolysis, endothelial dysfunction and platelet hyper-reactivity [3].

**Fig. 1:**
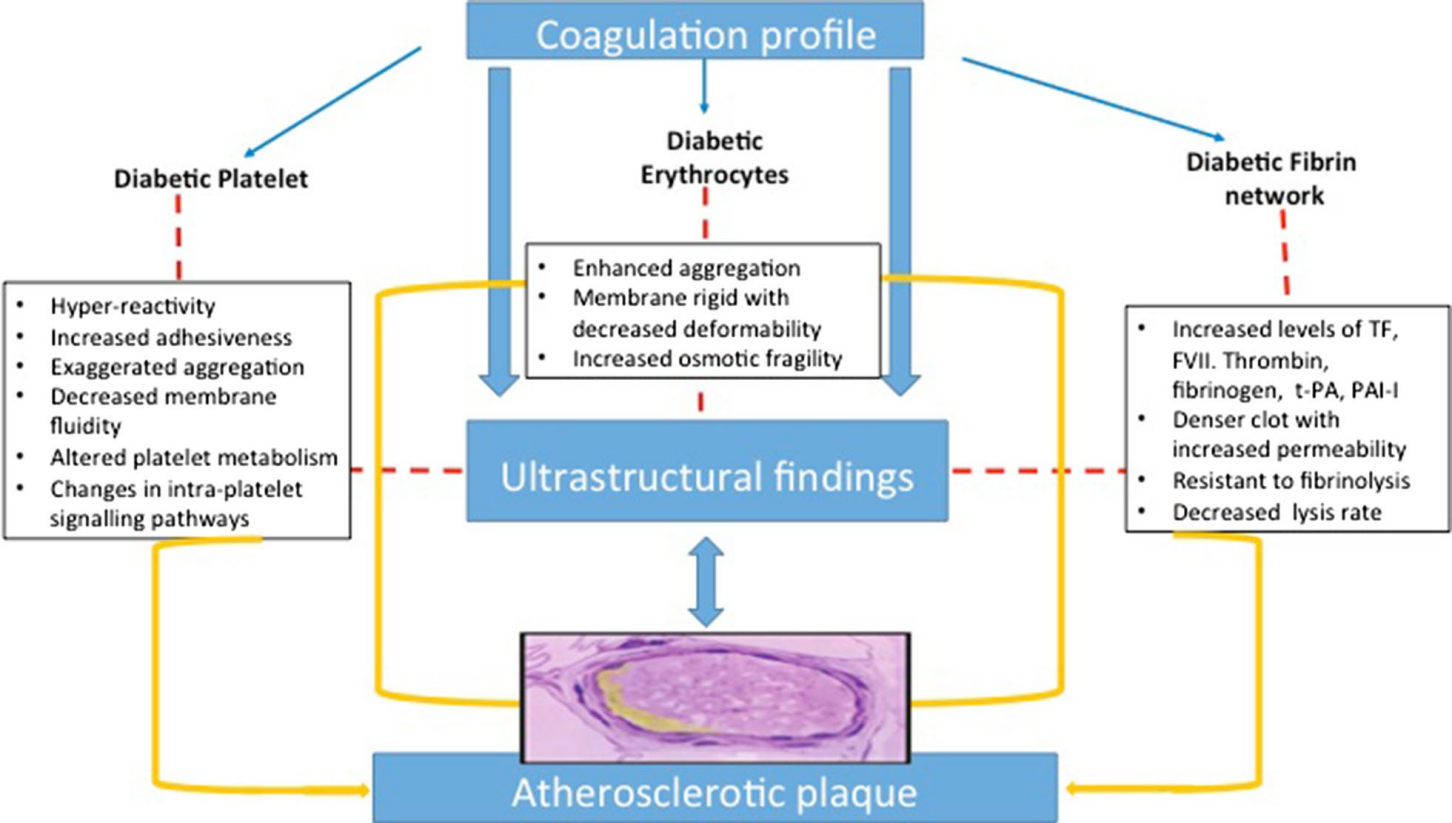
Outline of the review: cells involved in coagulation are described with characteristics thereof which contribute to atherosclerosis. Ultrastructural findings in all 3 cell types are then reviewed (*TF* tissue factor, *FVII* factor VII, *t-PA* tissue plasminogen activator, *PAI-I* plasminogen activator inhibitor I).

All stages in the pathophysiology of plaque formation with atherosclerosis are widespread and accelerated in type 2 diabetes. This is attributed to the imbalance in endothelial damage and repair mechanisms that are usually exhausted. The plaque is made up of erythrocytes, fibrin fibres and platelets [1]. Plaques are more susceptible to rupture and commonly referred to as “vulnerable plaque” syndrome. In particular, hyperglycaemia causing platelet activation, the increase in fibrinogen and hypofibrinolysis related to insulin resistance, all play a significant role in the development of angiopathy [4]. One research method that is used to study platelet structure and activation, is electron microscopy. This review defines ultrastructural findings in diabetic platelets, fibrin network and erythrocytes that can contribute to accelerated atherosclerosis. The outline of the review is highlighted in Fig. 1. The next paragraphs focus on structure and function of platelets, fibrin networks and erythrocytes in relation to type 2 diabetes.

## Review

### Platelets

An important element postulated in the pathogenesis of the prothrombotic state in diabetic patients is platelet hyper-reactivity. The prothrombotic condition that is characteristic in diabetes, in turn, can be ascribed to the following factors: (1) increased coagulation, (2) impaired fibrinolysis, (3) endothelial dysfunction and (4) platelet hyper-reactivity [3, 5]. In diabetes, several mechanisms contribute to platelet dysfunction, such as those due to hyperglycaemia, insulin deficiency and insulin resistance, associated metabolic conditions and other cellular mechanisms [6]. Even though platelets perform multiple functions, one of their primary functions is to respond to blood vessel injury by utilizing some of its unique characteristic like changing shape, secreting granule contents and aggregating to form a platelet clot. Secondary functions include: maintenance of vascular tone, inflammation, host defence and tumour biology. Two major storage granules in platelets are α- and dense granules. Alpha-granules are most abundant and contain proteins essential for platelet adhesion [7], while the dense granules function to recruit additional platelets to sites of vascular injury. Dense granules store molecules that are secreted upon platelet activation. Contents of dense granules include substances such as catecholamine, serotonin, calcium, adenosine 5′-diphos-phate and adenosine 5′-triphosphate [8].

Like with all cells, the plasma membrane lies beneath the outermost layer and its main component is a phospholipid bilayer in which cholesterol, glycolipids and glycoproteins are embedded. Unlike erythrocytes, platelets present these molecules on their surface. The organization of the phospholipids between the inner and outer leaflets is asymmetrical and this regulates coagulation. There is an abundance of negatively charged phospholipids in the inner leaflet of the plasma membrane which maintains the platelet surface in a non-procoagulant state [9]. The phospholipids contribute to coagulation by stimulating the coagulation factor X to Xa and prothrombin to thrombin, both integral steps in the coagulation cascade [10]. Other protein components of the resting platelets include CD36, CD63, CD9 and GLUT-3 [7]. These are platelet activation markers and Eibl et al. found that subjects with type 2 diabetes exhibit increased expression of CD31, Cd36, Cd49b, CD62P and CD63 [11]. Research has shown that enhanced platelet activation, aggregation and increased expression of CD63 and CD62 contribute to atherosclerosis and thrombosis in diabetes [12]. A cross section of the discoid platelet also reveals that the platelet membrane is densely packed with highly specific surface receptors that tightly controls signal-dependent platelet activation and may modify α-granule release to coagulation, inflammation, atherosclerosis, antimicrobial host defence, angiogenesis, wound repair or malignancy [7].

Insulin resistance is a common finding in subjects with type 2 diabetes as are the complications in the macrovascular and microvascular circulation. The insulin resistance is responsible for numerous alterations both at the metabolic and cellular levels. Included in the target tissues of insulin, in particular, the cellular systems that are affected is the endothelial cells, platelets, monocytes and erythrocytes [13]. Insulin’s action on platelets is to sensitize platelets to the inhibitory effects of prostacyclin and nitric oxide on aggregation and to diminish the proaggregatory properties of agonists such as prostaglandin E1, and E2. During platelet aggregation and activation, mechanisms like phospholipase C-induced hydrolysis of inositol phospholipids and opening of ion channels are activated. This results in various physiological responses by inducing changes in the phosphorylation state, activity of enzymes and structural properties of key platelet proteins [13].

#### Structural abnormalities found in diabetic platelets

Previous research has shown that diabetic platelets are characterised by increased adhesiveness and exaggerated aggregation. An array of mechanisms for the platelet hyper-reactivity has been postulated; decreased membrane fluidity, altered platelet metabolism (impaired calcium and magnesium homeostasis), elevated glycoprotein receptors, increased thromboxane A2, non-enzymatic glycation of surface proteins, enhanced generation of reactive oxygen species and decreased antioxidants with decreased prostacyclin and nitric oxide [2, 14].

**Fig. 2:**
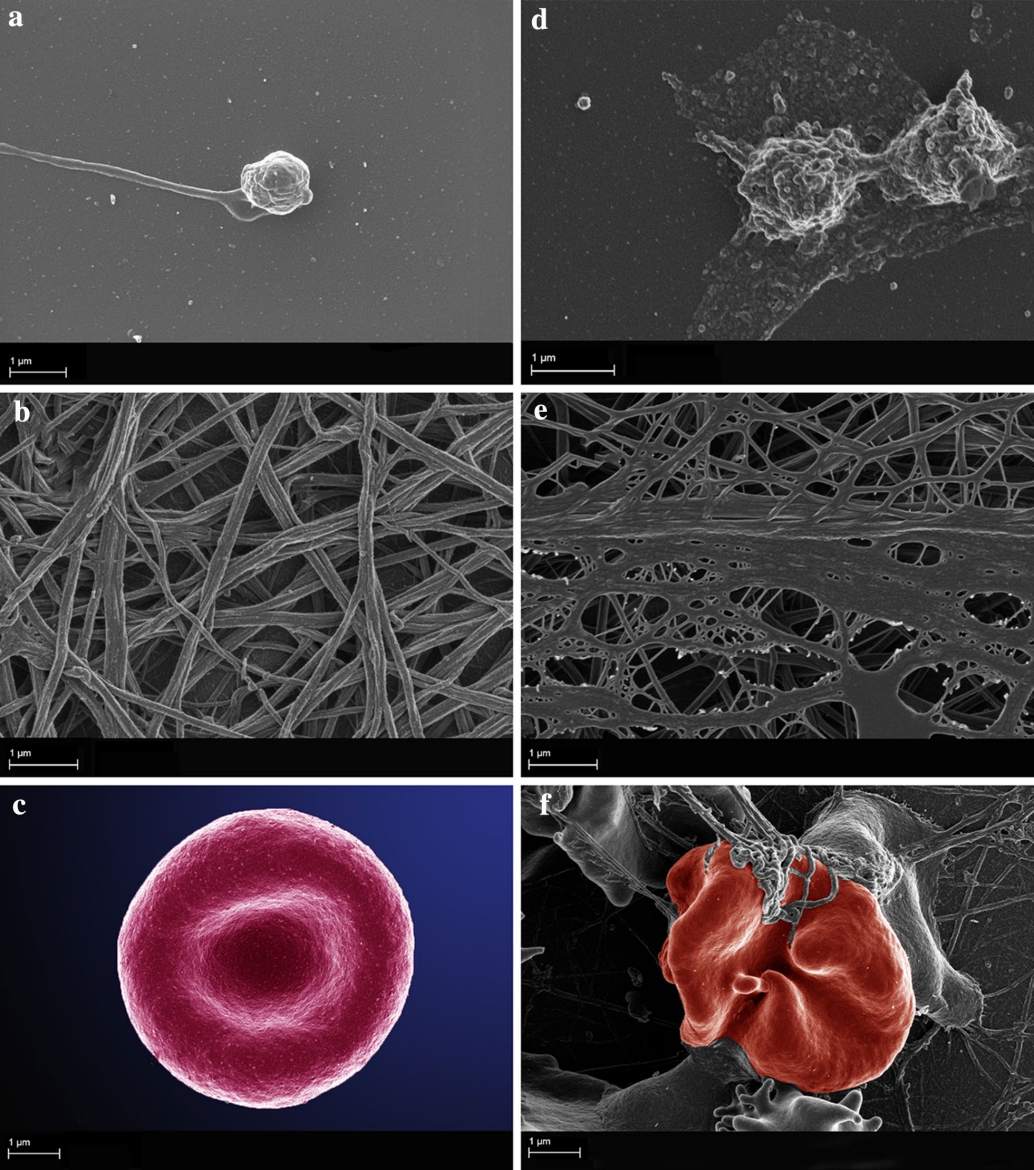
SEM of platelets, fibrin networks and erythrocytes. **a**–**c** Taken from healthy individuals. **d**–**f** Taken from diabetic subjects. **a** Control platelet showing extended pseudopodia. **b** Major fibres and minor fibres of fibrin network, in a healthy control. **c** Erythrocyte of a healthy individual showing the typical morphology. **d** Diabetic platelets showing blebbed morphology. **e** Fibrin network of a diabetic subject showing dense matted deposits. **f** Diabetic erythrocyte entrapped in atypical fibrin fibres.

Our research team has looked at the ultrastructure of platelets and fibrin networks in diabetic patients and confirmed a changed platelet membrane ultrastructure [15]. In our studies, platelets seemed shrunken and the membranes showed blebbing [15]. Barely no pseudopodia were seen, which normally develop spontaneously from platelets, as can be seen in control platelet. This blebbed morphology is typical of apoptosis as shown in Fig. 2d [15]. Due to the blebbed morphology, suggesting apoptosis, the integrity and surface of the platelet membrane is impaired, implying functional impairment. The finding of membrane blebbing is critical as it may cause an increase in microparticles in diabetes. Microparticles are membrane-coated vesicles that originate by budding from their parental cells upon activation or apoptosis [16]. Cell-derived microparticles support coagulation and inflammation and they may be involved in accelerated atherosclerosis in diabetic patients [17].

### Fibrin networks

There is an increased prevalence of atherothrombotic complications in subjects with diabetes. Prominent features contributing to premature atherosclerosis in this group include: increased platelet reactivity and activation of coagulation factors with associated fibrinolysis [18]. During blood vessel injury, the normal physiological response is that fibrin is deposited at the atherosclerotic lesion. Thus the structure of the deposited fibrin has become significant and is viewed as a probable risk factor for increased affinity for cardiovascular events in subjects with atherosclerosis [19]. Investigating ultrastructural morphological changes in activated platelets, as well as that of fibrin networks, is emerging as an important tool when studying different medical conditions. An aetiologic factor postulated to be causing ultrastructural changes in fibrin networks is abnormalities in the coagulation process [20, 21].

#### The role of coagulation proteins in the coagulation cascade

The coagulation cascade includes both clot formation and fibrinolysis. Coagulation proteins play significant roles in both the processes. Diabetic subjects are known to have higher levels of circulating tissue factor (TF), FVII (factor VII), thrombin, fibrinogen, tPA (tissue plasminogen activator) and PAI-I (plasminogen activator inhibitor-I) [18]. TF initiates the thrombotic process with the ultimate production of thrombin which is crucial for the conversion of fibrinogen to fibrin. The increased TF levels in diabetes are under the control of both glucose and insulin. In fact, the two controlling factors tend to have an additive effect [22]. Another mechanism implicated for the elevated TF levels is through the formation of advanced glycation end products and reactive oxygen species [23]. During the process of plaque rupture TF/FVII complex is formed. Together with the underlying platelet stimulation, this complex activates different coagulation factors with the ultimate generation of thrombin [18]. Like TF, FVII is also elevated in subjects with diabetes and in those with the metabolic syndrome [5]. Early work has shown that FVII coagulant activity has been associated with fatal cardiovascular events and more importantly, increased FVII coagulant activity is directly correlated with raised blood glucose [18].

In both type 1 and type 2 diabetes thrombin generation is enhanced [22]. The hyper-glycaemia found in diabetic subjects, is the culprit causing increased thrombin production and when the hyperglycaemia is controlled, thrombin production is reduced, proving the prothrombotic nature of hyperglycaemia. [24] High concentration of thrombin results in altered clot structures as they are denser and less permeable making them more resistant to lysis [22]. Fibrinogen, the precursor of fibrin is described as an independent risk factor for cardiovascular disease and is often used as a surrogate marker for cardiovascular risk [25]. High fibrinogen levels are known to have a predictive value in the setting of silent myocardial ischaemia especially in subjects with type 2 diabetes [25].

#### Description of fibrin networks in diabetes

Diabetic subjects have been shown to have altered fibrin network structures as illustrated in Fig. 2e, and this was first confirmed by Jörneskog and colleagues [26]. Their findings indicated that plasma clots from Type 1 diabetes subjects have reduced permeability suggestive of a more compact structure which is independent of the presence of microvascular complications. Using confocal microscopy techniques, Alzahrani and colleagues found that clots made from pooled plasma-purified fibrinogen in diabetic and insulin resistant subjects have a more compact fibrin network structure compared with controls, supporting earlier findings [18]. Figure 2b, e compares fibrin fibres of a healthy individual and that of a typical diabetic individual. Healthy fibrin fibres show individual fibres (Fig. 2b) while fibrin fibres in diabetes have thickened masses of fibres with a netted morphology as indicated Fig. 2e, giving the appearance of dense-matted net. This pattern is suggestive of a systemic inflammatory profile [15] and may contribute to the hypofibrinolysis which is a frequent and significant feature of diabetes.

Mechanisms involved in changing the fibrin network architecture in diabetes cause both quantitative and qualitative changes. Hyperglycaemia and insulin resistance induce qualitative changes as a result of increased glycation and oxidation while quantitative changes are associated with elevated levels of TF, thrombin, fibrinogen and PAI-1 as discussed above. The final end result is that the clot exhibits a denser structure and resistance to fibrinolysis [18]. Clots formed at high fibrinogen concentrations show unique properties and includes: thin fibres, reduced pore size and increased tensile strength; also this clot is degraded at a lower rate by plasmin [27].

The first intervention trial by Pieters and co-workers, investigated the effect of glycaemic control on fibrin network structures of type 2 diabetic subjects using isolated fibrinogen [28]. A variety of parameters were measured to highlight their findings and included, fibrinogen glycation, clot permeability, turbidity measurements, fibre diameter, visco-elastic properties, lysis rate and effect of fibrinogen glycation on FXIIIa cross-linking. Results obtained were as follows:

- Fibrinogen glycation: higher level of fibrinogen glycation among the diabetic group with a significant decrease upon achieving glycaemic control.
- Clot permeability: this parameter reflects the clot structure, specifically the average pore size. This was increased in the diabetic subjects and a significant correlation between permeability and HbA1c was also proven.
- Turbidity measurements: analysis of this parameter results in turbidity curves which are used to characterize the kinetics of polymerization and clot structure. The slope of increase in turbidity, representative of lateral aggregation was higher in the diabetic group.
- Fibre diameter: this was measured using scanning electron microscopy (SEM), and the median fibre diameter of the clots from the diabetics and controls did not differ.
- Viscoelastic properties: were similar between the controls and diabetics however, there was a lower proportion of inelastic component in the fibrin clots of the diabetic subjects.
- Lysis rate: the diabetic subjects had a lower lysis rate.
- Effect of fibrinogen glycation on FXIIIA cross-linking: No differences were detected among the diabetic and control groups.

Subjects with diabetes have higher than normal tissue factor levels. Of significance is that tissue factor activity is controlled by both insulin and glucose. Another stimulating factor for the synthesis of tissue factor particularly in diabetes is glycation end products and reactive oxygen species (ROS). Also the elevated production of thrombin in diabetic subjects has a direct effect on the clot formation, structure and stability. The final product is thus a denser, less permeable clot which is more resistant to lysis. Linking the concept of diabetes as a prothrombotic state and inflammation is the elevated cytokine, IL-6 which stimulates the hepatocytes to produce more fibrinogen. Increased production of fibrinogen by hepatocytes is also a common finding in insulin resistance [29].

### Erythrocytes

It is no surprise that just like platelets and fibrin networks, erythrocytes have been shown to play a role in coagulation as well. This is in striking contrast to their traditional physiological role of oxygen transport. In fact, emerging evidence suggests that erythrocytes enhance functional coagulation properties and platelet aggregation [30]. Also, erythrocytes have been localized in coronary atherosclerotic plaques [31]. Among the spectrum of haematological abnormalities in diabetes, erythrocyte aggregation and decreased deformability of erythrocytes feature strongly [32]. More importantly, they are implicated in the pathogenesis of microvascular complications of diabetes. The adverse effects of glucose manifests in multiple ways: rearrangement of erythrocyte membranes, defects in haemoglobin oxygen binding activity, alterations of mechanical features of the membrane and general aspects of the cell as well [33]. This is attributed to the prothrombotic nature of the erythrocytes as they increase blood viscosity and forcing platelets towards the vessel wall. Thus the integration of erythrocytes into a fibrin clot has an influence on clot structure and its mechanical properties [34–36].

### Diabetic-induced changes in the erythrocytes

#### Alterations in erythrocyte structure

In diabetic subjects the erythrocyte membrane becomes rigid and non-deformable. A decrease in the cholesterol to phospholipid ratio is responsible for this abnormality. Not only is the cholesterol component of the membrane increased but there is a four-fold increase in the phospholipids concentration, which results in a significant decreased ratio [37]. Of interest is that the proportion of membrane cholesterol is increased and has been predicted to contribute to instability of atherosclerotic plaque [38, 39]. Cytoskeletal proteins, in particular, beta spectrin, ankyrin and protein 4.1 are heavily glycosylated [40]. Disturbances in ionic balance is attributed to the lowered Na^+/^K^+^—ATPase activity and this leads to complications such as increased serum and intra-erythrocyte sodium and serum potassium in diabetic subjects. This also results in an increase in the cell size and increased osmotic fragility which contributes to the development of microvascular complications [41]. Elevated fibrinogen and glucagon are common findings in uncontrolled diabetes [42]. Oxidative stress plays a role in causing increased membrane lipid peroxidation and this may lead to abnormalities in composition and function. Enhanced levels of malondialdehyde (an indicator of lipid peroxidation) [43] and reduced levels of glutathione and membrane –SH group are features of the diabetic erythrocyte [44].

Despite absent mitochondria in the erythrocytes, they still depend on glucose as their energy source. However, in a hyperglycaemic environment, glycosylation of haemoglobin takes place which creates oxidative stress thereby making the cellular components of the erythrocyte more vulnerable [45]. One of the functions of a membrane is to provide protection and this includes against oxidative damage as well. However, in diabetic subjects, lipid peroxidation causes structural damage to the membrane with a subsequent decrease in the cell deformability and fluidity. SEM and AFM findings in the study by Buys et al., confirmed the correlation between the cytoskeletal protein and lipid layer damage with the ultrastructural roughness of the erythrocyte membrane found with AFM [46]. As mentioned previously, evidence points to glycosylation of cytoskeletal proteins and oxidative damage of spectrin [47].

#### Alterations in erythrocyte aggregation and deformability

The property of aggregation is guided by the composition of erythrocyte membrane and plasma proteins fibrinogen and globulin. When fibrinogen levels are increased and albumin is decreased, aggregation is enhanced. A mechanism that favours increased tendency of erythrocyte aggregation is the decreased ionic charge of the membrane. Microscopic examination of the erythrocyte aggregate reveals an increase in aggregate shape and size when compared to healthy controls. The parameter erythrocyte deformability unlike aggregation is modified by the composition of the membrane, cytoplasmic contents and age of the erythrocytes [42]. It is the measure of the ability of the cells to deform under applied shear stress. [47] Advanced technological measurements confirm that in diabetes, deformability is significantly decreased. This abnormality is attributed to the specific changes in the membrane structure. The consequence of altered deformability is the increase in blood viscosity which can lead to increase in shear stress on the endothelial wall [42]. Investigation of diabetic erythrocytes with cardiovascular complications is associated with lowered membrane fluidity when compared to healthy controls. The diffusion of protein and lipid molecules within the membrane is known as membrane fluidity and is dependent on the presence of saturated and polyunsaturated fatty acid [48]. The increased tendency of erythrocyte from subjects with diabetes to adhere to cultured human vascular endothelial cells was confirmed by research performed by Wali et al. Their results suggest that a possible mechanism for the latter is a loss of lipid asymmetry and or less ordered packing in the outer leaflet of the diabetic erythrocyte membrane [49].

Diabetes, a multifactorial disease has a marked effect on the rheological and electrical properties of the erythrocyte. The erythrocyte is commonly described as more rigid than normal with a reduced deformability. However diabetes also causes profound changes on the ultrastructure of erythrocytes, fibrin networks and platelets. Pretorius and co-workers showed a changed morphology in type 2 diabetic erythrocytes using the scanning electron microscope (SEM), which showed elongated cells forming extended projections that twist around spontaneously formed fibrin fibres as indicated in Fig. 2f. The causative role of iron overload and subsequent non-enzymatic fibrinogen polymerization on altering the ultrastructure of erythrocytes in type 2 diabetes, has been detailed by Lipinski et al. [50, 51].

In the process of a thrombotic event many parameters (which are increased) play a role, however, increased levels of fibrinogen feature strongly. This is of significance as it causes abnormal fibrin fibre formation visible as dense matted deposits (DMD) and the resulting coagulum causes blood cells to change shape and to be trapped in the abnormal mesh [52]. An array of inflammatory diseases, including type 2 diabetes mellitus are associated with increased fibrinogen levels and hypercoagulability which results in markedly changed fibrin morphology, DMDs [53]. DMDs may therefore reflect a hypercoagulable profile. Of note is the close link between this hypercoagulability of fibrin and involvement of erythrocytes [54, 55]. Our research team has also shown that when fibrin clots abnormally, erythrocytes are entrapped more tightly inside the clot as indicated in Fig. 2f [51, 56]. The factors of both erythrocyte aggregation and fibrinogen interactions cause a changed viscosity which impacts on the optimal functioning of erythrocytes. It is thus difficult to ignore the influence of increased blood viscosity and increased fibrin concentration, not only in their role as strong predictors of cardiovascular diseases but also as important factors in the development of atherosclerosis [56–59].

## Conclusion

Platelet dysfunction poses an increased risk for thrombotic vascular events. The significance of platelet abnormalities in the atherothrombotic process has been highlighted by the use of antiplatelet drugs that form part of the therapeutic regime in reducing cardiovascular risk [2]. The multifactorial aetiologies for platelet dysfunction is beyond the scope of this review, however, diabetes and the associated hyperglycaemia cannot be ignored. Hyperglycaemia induces a hypercoagulable condition and contributes to micro- and macrovascular disease. Studies have shown that optimal control of both fasting and post-prandial glucose levels will reduce the impact. It remains to see if the latter will improve morphological findings of the platelet ultrastructure in diabetic subjects. Suppression of fibrinolysis and increased fibrinogen are among the group of haemostatic abnormalities in type 2 diabetes. Increased fibrinogen adds to the burden of cardiovascular risk by increasing blood viscosity, increasing the size of the clot, tissue deposition is increased and stimulation of atherosclerosis and vascular thickening. Erythrocytes too contribute to the high incidence of atherosclerotic diseases in diabetes partly due to the association of abnormalities of erythrocyte composition and rheological function with increased oxidative stress.

The combination of blebbed platelets, abnormal fibrin in the form of dense matted deposits and changed erythrocytes morphology creates a thrombotic risk cluster which underpins the development of cardiovascular disease. Analysis of ultrastructural findings in diabetic platelets, fibrin network and erythrocytes can reveal significant findings which may add to a better understanding of the pathogenesis of atherosclerosis and thrombosis.
